# Issues in Managing Hurthle Cell Carcinoma of Thyroid: A Case Report

**DOI:** 10.7759/cureus.1167

**Published:** 2017-04-14

**Authors:** Patricia Tai, Martin Korzeniowski, Evgeny Sadikov, Kurian Joseph, Angus Kirby, Jon Tonita, Aamer Mahmud

**Affiliations:** 1 Department of Radiation Oncology, Allan Blair Cancer Center, University of Saskatchewan; 2 Department of Radiation Oncology, BC Cancer Agency, Kelowna, BC; 3 Department of Radiation Oncology, Cross Cancer Center, University of Alberta; 4 Department of Pathology and Laboratory Medicine, Pasqua Hospital, Regina, Saskatchewan; 5 Department of Epidemiology, Saskatchewan Cancer Agency, Regina, Saskatchewan; 6 Oncology, Kingston General Hospital and Hotel Dieu Hospital, Queens University

**Keywords:** hurthle cell, thyroid cancer, management, radioiodine, imaging, surgery, radiotherapy, diagnosis, pathology, chemotherapy

## Abstract

A 61-year-old woman noticed a right neck lump in October 2001. Fine needle aspiration showed follicular neoplasm, adenoma versus carcinoma. The ultrasound scan showed a solid mass of maximum dimension of 3.7 cm. She had a right thyroid lobectomy and isthmectomy in January 2002 (first surgery). The tissue specimen showed a 4.5 cm Hurthle cell carcinoma (HCC) with vascular invasion. There were no capsular invasion, extra-thyroidal extension, or margin involvement. A completion left lobectomy (second surgery) was performed two weeks later. Therefore the pathological stage is II (T3N0M0). She received adjuvant radioactive iodine ablation for residual thyroid tissue. By 2003, she developed local recurrence, which was resected (third surgery), followed by adjuvant external beam radiotherapy. Unfortunately, she developed further recurrence in the left main bronchus, as identified by Indium-111 Octreotide (Curium, Missouri, USA) and positron emission tomography-computed tomography PET-CT imaging in 2006. She underwent a left pneumonectomy (fourth surgery) in July 2006. In November 2007 she was found to have mediastinal recurrence which was treated with high-dose external beam radiotherapy. She initially responded but developed more local recurrence and a lung metastasis by 2011. She was treated with brivanib with ixabepilone, under a phase I clinical trial with mixed response. Her treatment was discontinued secondary to toxicity and she succumbed to her disease in 2012. This case report illustrates the natural history and clinical decision making for patients diagnosed with HCC of the thyroid. Specifically, we highlight the clinical issues surrounding the histopathological diagnosis, extent of surgical resection, radioiodine diagnostic imaging/ablative treatment, as well as external beam radiotherapy.

## Introduction

Hurthle cell carcinoma (HCC) of the thyroid, also called oncocytic carcinoma, is a rare form of differentiated thyroid cancer. Preoperative clinical, cytological, and genetic studies have not been shown to reliably discriminate between benign and malignant variants of Hurthle cell neoplasms (HCN) [1]. Therefore, histopathological analysis remains the gold standard for diagnosis; although, it may be apparent preoperatively if there is evidence of metastasis.

## Case presentation

A previously healthy 61-year-old woman noticed a right neck lump in October 2001. The ultrasound scan demonstrated a solitary 3.7 x 3.4 x 3.0 cm heterogeneous solid mass. The family doctor performed a fine needle aspiration in the office that showed follicular neoplasm, adenoma versus carcinoma. The depicted risk of malignancy by Bethesda system is 15-30% [2]. Table 1 summarizes her story.

**Table 1:** Summary of diagnostic tests and treatment of the case. CT, computerized tomography; ERT, external beam radiotherapy; Gy, gray – absorbed dose of radiotherapy; I, radioactive iodine; L, left; PET, positron emission tomography; R, right.

TIME	PATIENT SYMPTOMS	DIAGNOSIS METHOD	TREATMENT		
			Surgery	Radiation	Systemic
Oct 2001	R neck lump	Fine needle aspiration, ultrasound			
Jan 2002	-	Frozen section, final pathology	First surgery: R thyroid lobectomy & isthmectomy, Second surgery: L lobectomy.		
Apr 2002	-	I-123 scan		I-131 ablation, nine days later whole body scan	
Jan 2003	-	Non-contrast CT scan neck & chest. Whole body I-123 scan	Third surgery: excision of local recurrence	Adjuvant ERT	
Jan 2006	-	Serum thyroglobulin & antibody, Indium-111 octreotide & PET/CT scan	Fourth surgery L pneumonectomy for the L hilar mass and L lower lobe metastases, July 2006		
Nov 2007	-	CT scan		Palliative ERT	
Apr 2012	-	CT scan			Clinical trial: brivanib + ixabepilone

She had a right thyroid lobectomy and isthmectomy in January 2002 (first surgery) because the frozen section could not tell if the tumor was benign or malignant. The tissue specimen showed a 4.5 cm Hurthle cell carcinoma (HCC) with vascular invasion. There were no capsular invasion, extra-thyroidal extension, or resection margin involvement. A completion left lobectomy (second surgery) was performed two weeks later. The pathological stage was II: T3N0M0 due to the maximum tumor dimension of 4.5 cm (TNM 1997 Staging). The histological type was HCC and was confirmed with another pathologist in a tertiary center.

**Figure 1:**
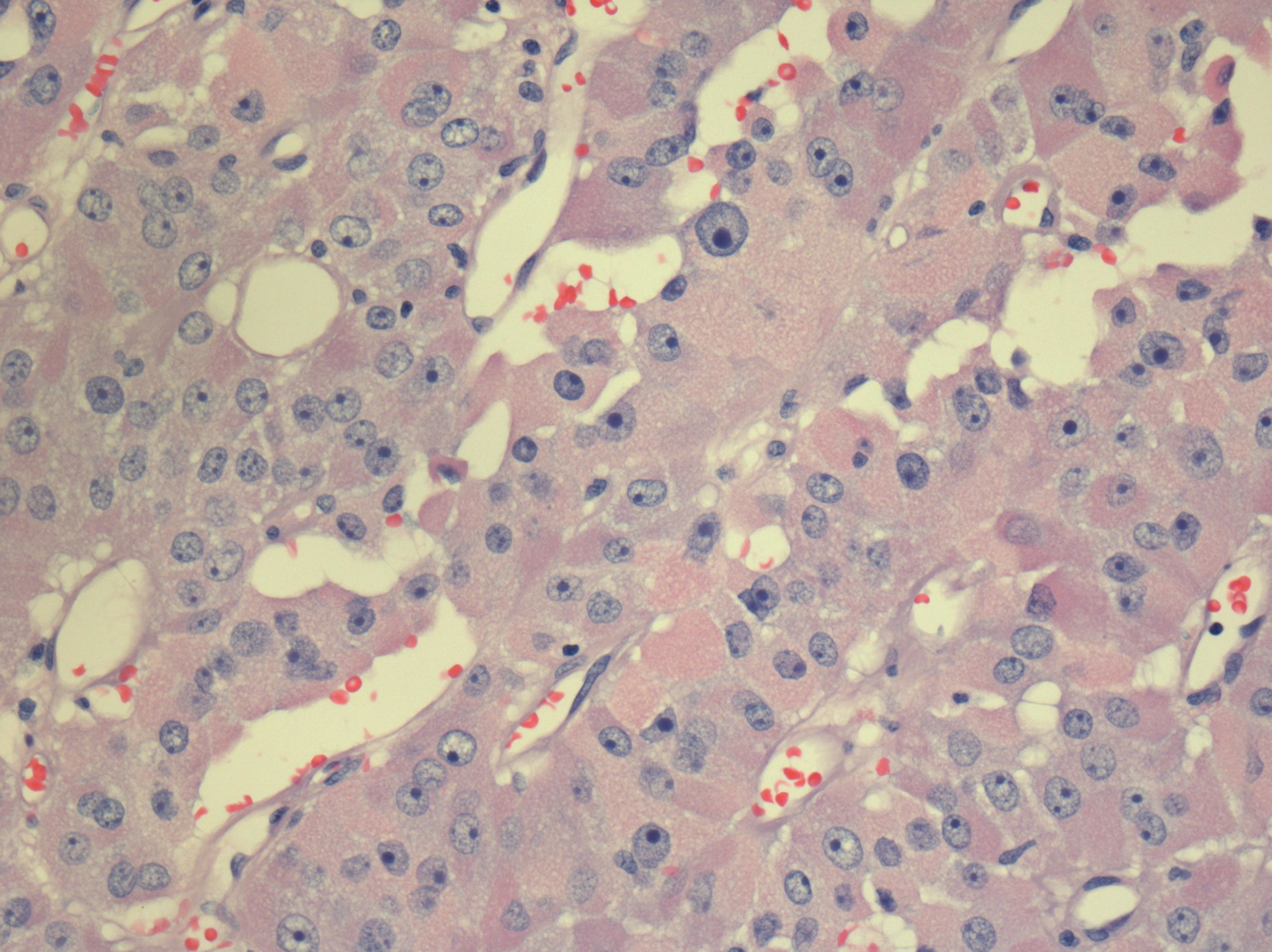
Pathology slide of the recurrent tumor.

As there was a significant amount of residual thyroid tissue by a radioactive Iodine-123 scan, ablation with 100 milliCurie of Iodine-131 was given in April 2002. Unfortunately by January 2003, a doctor felt a 0.6 cm lump in the right neck scar. There was no nodal or distant recurrence with a non-contrast computerized tomography (CT) scan. The Iodine-123 scan did not show any uptake of the radio-isotope in the neck or the rest of the body. The local recurrence in the right neck was locally excised in March 2003 (third surgery) followed by adjuvant radiation 58 Gray (Gy) in 29 treatments in June 2003. A follow-up radioactive iodine scan reported no uptake in the neck including the scan in November 2005. However, serum thyroglobulin gradually rose with time: November, 2003: 4.0; June, 2005: 76.8; November, 2005: 642.2 pmol/L (the upper limit of normal should be <5 pmol/L after thyroid ablation). Serum thyroglobulin antibodies were not present. An Indium-111 Octreotide scan (Curium, Missouri, USA) in January 2006 showed uptake at the level of the bifurcation of the left main bronchus. While on thyroxine, she underwent a positron emission tomography/ computerized tomography (PET/CT) scan in March 2006, which showed a 2.2 x 2.7 cm left hilar mass and a small 8 mm pre-carinal node. The lung fields were clear. No other abnormalities were found. She saw our thoracic surgeon who felt that a left pneumonectomy was required to remove the left hilar mass, which grew to 3.3 x 2.6 cm, and a new 1.2 x 0.6 cm left lower lobe metastasis seen in the June 2006 CT scan. She then underwent left pneumonectomy in July 2006 (fourth surgery). A pathology slide of the lung specimen is shown in Figure 1. The tumor cells are in solid sheets having high nuclear/cytoplasmic ratio, hyperchromatic nuclei, and prominent nucleoli with granular eosinophilic cytoplasm.

**Figure 2:**
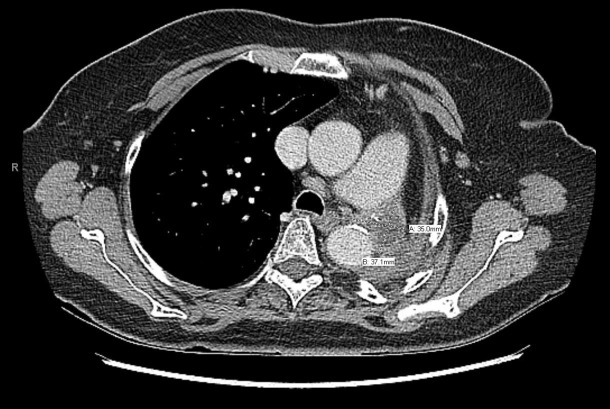
Computerized tomography scan of the chest in December 2011 showing that the left mediastinal soft tissue recurrence around the aortic arch regrew to a size of 3.5 x 3.7 cm.

By November 2007, a CT scan detected a 2 cm left mediastinal recurrence, but the patient initially declined treatment due to fear of complications. Finally, she agreed to treatment by palliative radiotherapy 60 Gy in 30 fractions to the left upper chest for the mediastinal recurrence, which was finished in October 2008. There was no overlap with the previous radiotherapy fields. The left mediastinal soft tissue recurrence around the aortic arch initially responded in follow-up CT scans, but began to regrow by December 2011 to 3.5 x 3.7 cm (Figure 2). In addition, there was a separate nodule in the right middle lobe that had grown to 1.5 x 1.1 cm.

In April 2012 she participated in a phase I trial of target therapy (brivanib) with a new chemotherapy (ixabepilone). She was admitted due to bladder infection and febrile neutropenia. A CT scan in May 2012 showed that the right middle lobe lesion had been reduced to 1.1 x 0.7 cm. The mediastinal mass was stable in appearance. She declined further treatment due to treatment toxicities.

By November 2012, the CT scan showed that the right middle lobe mass had grown to 1.9 x 2.4 cm and the mediastinal mass around the aortopulmonary window to 7.3 x 5 cm. There were also a new 3 x 2.5 cm right peribronchial mass and a 4 x 4.9 cm infra-carinal mass. She died of progressive disease by early December 2012.

## Discussion

The case report illustrates the decision-making process in HCC. HCC is a less common type of differentiated thyroid cancer. It has distinct adverse features: higher rate of regional lymph node metastases, recurrences and cause-specific mortality, all shown in this case [3]. Four management issues will be addressed below.

The first issue is about the difficulty to separate HCC from other benign or malignant thyroid neoplasms preoperatively or even by frozen section like our case. Hurthle cells (HC), or oncocytes, are large cells found in the thyroid gland and elsewhere, and are characterized by abundant, granular, eosinophilic cytoplasm which is a consequence of the accumulation of altered mitochondria. Pathological differential diagnoses include medullary carcinoma and papillary thyroid carcinoma. Workup typically involves a fine needle aspiration biopsy (FNAB). The Bethesda system for reporting thyroid cytopathology classifies HCC in the diagnostic category of “follicular neoplasm or suspicious for a follicular neoplasm” [2]. Histological features of capsular and vascular invasion have been used for many years as a sign of malignancy. Our case has vascular invasion in the final pathology report. Since it is undesirable to over-treat benign HCN, there is growing research in genetic profiling. The genetic abnormalities identified in HCN include aneuploidy, H-ras mutations and allelic alterations and include alterations in mitochondrial DNA (or genes) [4]. The cellular proliferation index (Ki67) was found to be higher in HCC. It may help as a diagnostic marker of malignancy in HCN cytology. However, larger studies are needed to validate these findings and, until then, testing for genetic alterations in HCC tumors remains primarily investigational [4].

The second issue is about the extent of surgical resection. Our case had completion thyroidectomy very soon after the pathology report came back as HCC. An ipsilateral neck lymphadenectomy is recommended for clinically/ radiologically/ biopsy-proven lymphadenopathy. Like our case, all patients should have a radioiodine scan three to four months after surgery. The iodine-123 scan was a diagnostic scan to see any residual thyroid tissue. Since there was a lot of residual thyroid tissue, she had I-131 ablation with 100 mCi, followed by a whole body scan nine days later.

The third management issue is on imaging and the role of radio-iodine diagnostic scan and treatment. The 2016 National Comprehensive Cancer Network (NCCN) guidelines recommended consideration of radio-iodine therapy based on pathological findings and postoperative serum thyroglobulin levels [5]. Except for low-risk cases, remnant thyroid could be ablated with iodine-131 to facilitate the use of serum thyroglobulin for surveillance. This will ablate the residual thyroid tissue, identify and treat known metastases. The current recommendation advocates its use in HCC patients with tumors >2 cm or those with nodal and distant metastases.

In patients with a negative radioiodine scan and rising serum thyroglobulin, PET/CT can be ordered. A meta-analysis of seven trials showed an improved detection rate of recurrence/metastasis by either thyroxine withdrawal [6] or recombinant thyroid stimulating hormone (rTSH) injection. Our patient underwent the PET/CT while on thyroxine.

The last issue is on the role of external beam radiotherapy. Our center employed this for gross postoperative residual disease and in this case for adjuvant and salvage treatments. In the modern era, our center also arranges radiofrequency ablation or stereotactic body radiotherapy for limited small lung metastases. Other salvage treatments include ethanol ablation, radioiodine ablation, external radiotherapy, radioisotope and systemic treatment. Our patient had external radiotherapy and systemic treatment.

## Conclusions

The reported case illustrates the management of this rare cancer. The patient presented with a neck mass, which was investigated with an ultrasound scan. We tried to establish the pathological diagnosis by a fine needle aspiration biopsy and a frozen section of the resected tissue, both of which could not confirm malignancy. The detailed examination found vascular invasion and concluded with the diagnosis of HCC. We performed radioactive iodine ablation, multiple surgeries, external radiotherapy and finally enrolled her into a clinical trial of systemic therapies due to lack of options. Despite our impeccable follow-ups, assessment, and treatments, she suffered multiple recurrences in different locations. Sadly, we lost her after 11 years of the cancer battle. The discussed strategic diagnosis and treatment plans could be used for other cases with successful outcomes. Thus, these approaches will provide insights to the medical and clinical science community.

## References

[1] Cannon J (2011). The significance of Hürthle cells in thyroid disease. Oncologist.

[2] Cibas ES, Ali SZ (2009). The Bethesda system for reporting thyroid cytopathology. Am J Clin Pathol.

[3] Haugen BR, Alexander EK, Bible KC (2015). Association management guidelines for adult patients with thyroid nodules and differentiated thyroid cancer: the American Thyroid Association Guidelines Task Force on thyroid nodules and differentiated thyroid cancer. Thyroid.

[4] Donatini G, Beaulieu A, Castagnet M (2016). Thyroid Hürthle cell tumors: research of potential markers of malignancy. J Endocrinol Invest.

[5] (2017). Clinical practice guidelines in oncology (NCCN Guidelines®) thyroid carcinoma. http://www.nccn.org/professionals/physician_gls/f_guidelines.asp.

[6] Ma C, Xie J, Lou Y (2010). The role of TSH for 18F-FDG-PET in the diagnosis of recurrence and metastases of differentiated thyroid carcinoma with elevated thyroglobulin and negative scan: a meta-analysis. Eur J Endocrinol.

